# Community‐led HIV testing services including HIV self‐testing and assisted partner notification services in Vietnam: lessons from a pilot study in a concentrated epidemic setting

**DOI:** 10.1002/jia2.25301

**Published:** 2019-07-19

**Authors:** Van Thi Thuy Nguyen, Huong TT Phan, Masaya Kato, Quang‐Thong Nguyen, Kim A Le Ai, Son H Vo, Duong C Thanh, Rachel C Baggaley, Cheryl C Johnson

**Affiliations:** ^1^ World Health Organization, Country Office Hanoi Vietnam; ^2^ Viet Nam Authority for HIV/AIDS Control Hanoi Vietnam; ^3^ Can Tho Provincial AIDS Centre, Vietnam Can Tho City Vietnam; ^4^ Thai Nguyen Provincial AIDS Centre Thai Nguyen City Vietnam; ^5^ National Institute for Hygiene and Epidemiology Hanoi Vietnam; ^6^ HIV Department World Health Organization Geneva Switzerland; ^7^ Clinical Research Department London School of Hygiene and Tropical Medicine London UK

**Keywords:** HIV, community, lay provider, self‐testing, partner notification, key and vulnerable populations, Vietnam

## Abstract

**Introduction:**

The HIV epidemic in Vietnam is concentrated in key populations and their partners – people who inject drugs, men who have sex with men, sex workers and partners of people living with HIV. These groups have poor access to and uptake of conventional HIV testing services (HTS). To address this gap, lay provider‐ and self‐testing and assisted partner notification (aPN) were introduced and delivered by the community. We explored the feasibility and effectiveness of implementing aPN as part of community testing services for key populations.

**Methods:**

Lay provider testing and self‐testing was started in January 2017, and targeted key populations and their partners. Since July 2017, aPN was introduced. HTS was offered at drop‐in houses or coffee shops in Thai Nguyen and Can Tho provinces. All self‐testing was assisted and observed by peer educators. Both in‐person and social network methods were used to mobilize key populations to test for HIV and offer HTS to partners of people living with HIV. Client‐level data, including demographic information and self‐reported risk behaviour, were collected on site by peer educators.

**Results:**

Between January 2017 and May 2018, 3978 persons from key populations were tested through community‐led HTS; 66.7% were first‐time testers. Of the 3978 clients, 3086 received HTS from a lay provider and 892 self‐tested in the presence of a lay provider. Overall, 245 (6.2% of tested clients) had reactive results, 231 (94.3%) were confirmed to be HIV positive; 215/231 (93.1%) initiated antiretroviral therapy (ART). Of 231 adult HIV‐positive clients, 186 (80.5%) were provided voluntary aPN, and 105 of their partners were contacted and received HTS. The ratio of partners who tested for HIV per index client was 0.56. Forty‐four (41.9%) partners of index clients receiving HTS were diagnosed with HIV, 97.7% initiated ART during the study period. No social harm was identified or reported.

**Conclusions:**

Including aPN as part of community‐led HTS for key populations and their partners is feasible and effective, particularly for reaching first‐time testers and undiagnosed HIV clients. Scale‐up of aPN within community‐led HTS for key populations is essential for achieving the United Nations 90‐90‐90 targets in Vietnam.

## 1 Introduction

In 2017, there were an estimated 250,000 people living with HIV (PLHIV) in Vietnam, a prevalence of 0.4% among adults aged 15 to 49 years 1. The majority of PLHIV are from key populations – people who inject drugs (PWID), men who have sex with men (MSM), female sex workers (FSWs) and their sexual partners. In 2017, there were an estimated 230,000 PWID, 170,000 MSM and 86,000 FSWs 2. Vietnam has made significant progress in expanding HIV testing services (HTS) as well as prevention services and provision of antiretroviral therapy (ART). In 2016, approximately 70% of PLHIV knew their status and 50% of all PLHIV were receiving ART in Vietnam 2. ART coverage in key populations such as PWID, MSM and FSWs in 2016 was 53.4%, 17.7% and 27.6% respectively 1.

Despite these gains, access to and uptake of HTS among key populations and their partners remain limited in Vietnam. In 2017, only 40% of FSWs, 62% of PWID and 65% of MSM with HIV had been diagnosed 2, likely due to low coverage and uptake of testing in these groups.

The Government of Vietnam has committed to achieving the United Nations 90‐90‐90 targets and efforts are being made to expand HIV testing 3, 4. However, stigma and discrimination, and inconvenient facility hours and locations hinder efforts to increase testing coverage among key populations. Innovative approaches are needed to address these barriers and reach key populations and their partners.

Assisted partner notification (aPN) services, where providers offer direct assistance to PLHIV to contact their sexual or drug‐injecting partners, are acceptable and highly effective for HIV case‐finding, and facilitating linkage to prevention and care services 5, 6, 7, 8, 9, 10. Several programmes in concentrated epidemics have successfully implemented aPN methods 11. Partner services among MSM have been well taken up when implemented through alternative testing sites, using social network approaches and offering opt‐out options and multiple follow‐ups 12, 13, 14. Other studies showed that aPN services increased HIV testing and linkage to care of previously unserved partners 15, 16. Qualitative investigation of preferences among FSWs suggests that aPN is particularly acceptable for contacting more “casual” and non‐primary partners 17 and can be affordable and cost‐effective, particularly when implemented by trained lay providers 18, 19, 20. When implemented within community‐led approaches or self‐testing, it can contribute to reaching more key populations, men, first‐time testers and an earlier diagnosis compared to facility‐based approaches 9, 21, 22, 23, 24, 25, 26.

Despite the benefits and successes, efforts to increase implementation and uptake of aPN remains low, particularly among key populations 6. Such low uptake and implementation are often linked to resistance due to negative perceptions, suboptimal implementation, unfriendly facilities and concerns about confidentiality or potential stigma and discrimination 27.

To address this gap, we conducted an implementation study to understand and assess the feasibility and effectiveness of implementing aPN as part of community testing services for key populations in Vietnam.

## 2 Methods

### 2.1 Pilot on partner notification within a context of lay provider and HIV self‐testing

**Figure 1:**
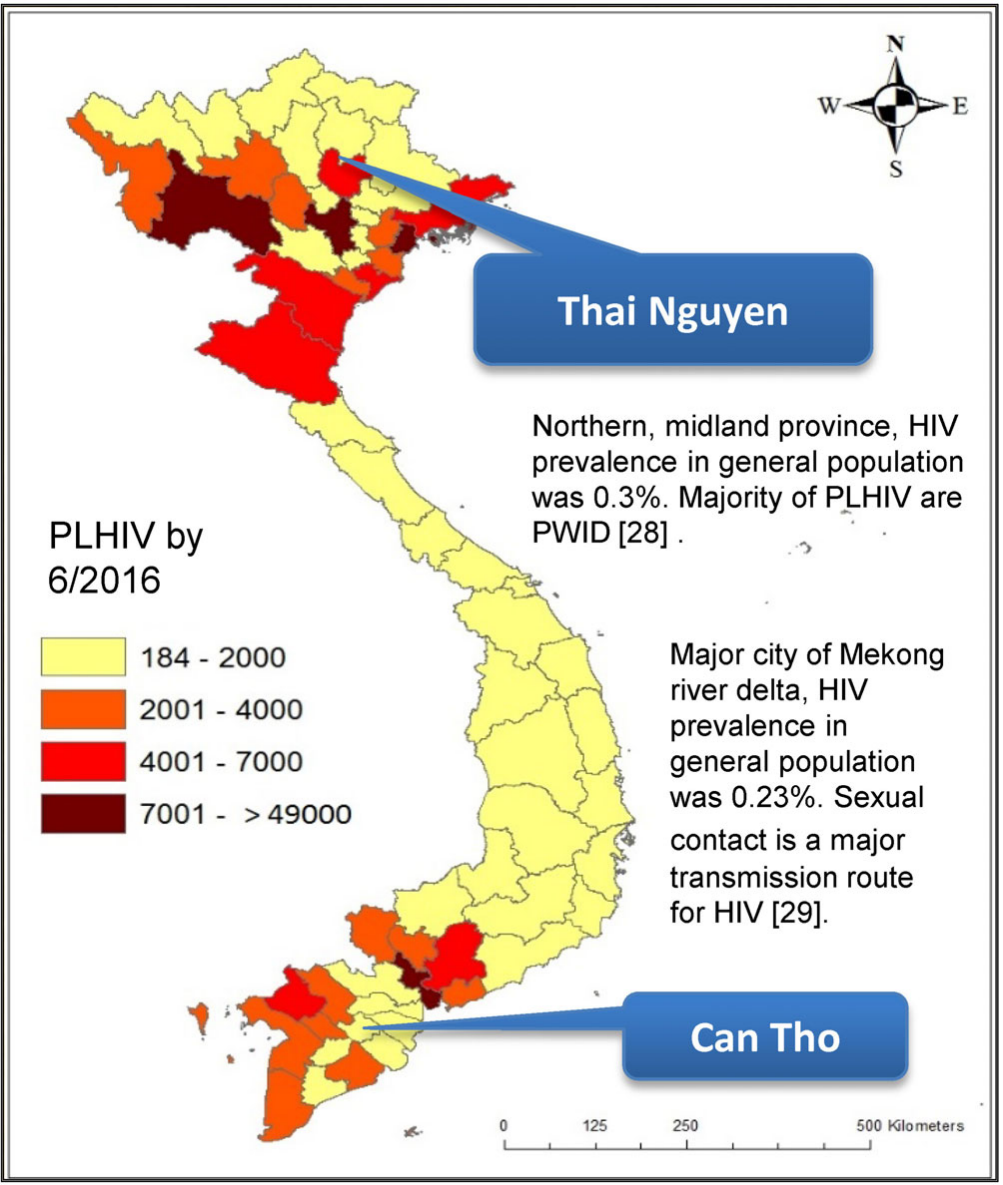
**Map of the HIV epidemic in Vietnam** PLHIV, people living with HIV; PWID, people who inject drugs.

Starting in January 2017, community‐led HTS, comprising lay provider‐delivered rapid testing, self‐testing and aPN, was delivered to key populations in one high‐HIV burden (Thai Nguyen in the north) and one medium‐HIV burden province (Can Tho in the south) (Figure 1). The major modes of transmission were injecting drug use in Thai Nguyen, and sexual transmission in Can Tho 28, 29. Community‐led HTS was implemented in a step‐wise manner. From January to May 2017, only lay‐provider testing and self‐testing with a blood‐based HIV rapid diagnostic test (RDT) was available. From July 2017 to May 2018, aPN services were additionally offered and self‐testing using oral fluid‐based HIV RDTs was also made available.

Lay providers, for example, peer educators who were MSM, PWID and FSWs without previous medical knowledge or experience of conducting HIV testing and outreach, were recruited and trained by the Provincial AIDS Centre (PAC) with technical assistance from World Health Organization (WHO). They were trained to conduct rapid testing, perform and demonstrate self‐testing, provide pre‐test information and post‐test counselling, and deliver aPN. Additional training on data collection and reporting was also provided and supervised by PACs. Peer educators received VND 800,000 (approximately US$ 35) per month for their time.

Peer educators used various approaches to mobilize key populations and their partners to test for HIV. Face‐to‐face outreach and small group discussions through existing networks were used to mobilize PWID and FSWs and reach their partners. Social media networks and dating apps were used to mobilize MSM and their partners. For instance, the information on HIV testing (time, date, and location of testing services) was posted in a Facebook MSM network in Can Tho named “Boy Love Can Tho” with more than 2400 members. In addition, lay providers also had their own Facebook groups to promote HIV testing and provide private chats with at‐risk individuals, including partners of HIV‐positive clients. In addition to Facebook, Zalo was also used to follow‐up clients or provide further counselling on testing and ART, as this is also a free internet call. Peer educators offered voluntary aPN to all clients with a newly confirmed HIV‐positive diagnosis using one of the following methods: provider referral (e.g. direct assistance from peer educator), dual referral (e.g. peer educator and client notify partner together) or client referral (e.g. client notifies partner alone). Often, they offered aPN during post‐test counselling when supporting linkage or adherence to treatment. The identity of index clients was kept confidential unless clients agreed to disclose his/her HIV status to their partners. No time restriction was set for when the index client and partner had sexual contact to be eligible for aPN.

As part of community‐led HTS, peer educators provided pre‐test information during mobilization and outreach prior to testing. Clients who agreed to test were given the option of testing themselves (self‐testing) or be tested by a peer (lay‐provider testing) using either a blood‐based RDT (SD Bioline HIV1/2 3.0 and SD Bioline HIV/Syphilis duo, Standard Diagnostics, Giheung‐gu,Yongin‐si, Korea) or an oral fluid‐based RDT (OraQuick^®^ HIV‐1/2; Orasure Technologies, Bethlehem, PA, USA). All HTS was delivered with a single RDT via a lay provider or a self‐test conducted in the presence of a peer educator.

**Figure 2:**
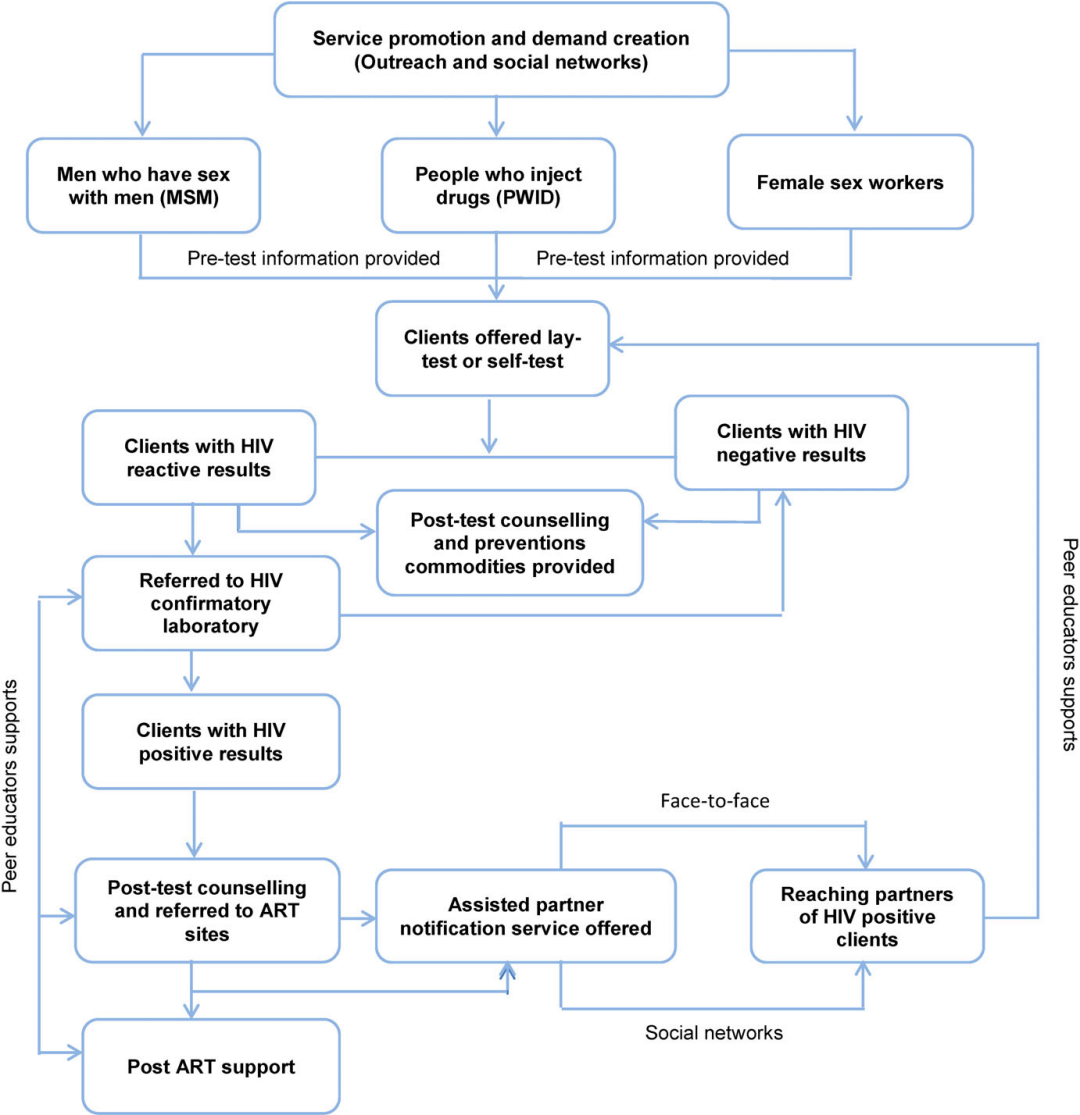
**Flowchart of community‐led testing, including HIV self‐testing and assisted partner notification services** ART, antiretroviral therapy.

Post‐test counselling was tailored to the needs of the client. Commodities for prevention, such as needles and syringes, condoms and lubricants, were provided to all clients according to their need. Clients with a negative test result were advised to retest in six months. Clients with a reactive result were counselled and supported by a peer educator, and referred for confirmatory testing. Those confirmed to have HIV positive received additional post‐test counselling by staff at the site of confirmatory testing. Peer educators then assisted them with enrolment in ART at an outpatient clinic. The possibility of social harm, including self‐harm and family conflict or violence, was discussed during the training. Clients with HIV were advised to contact peer educators if they faced any emotional challenges. Peer educators also conducted follow‐up visits with PLHIV to encourage linkage and document any social harm reported by clients, or observed by provincial coordinators and peer educators. An overall diagram of Community HTS – include HIVST and aPN cascade was described in Figure 2.

Staff from the PAC monitored and supervised peer educators, with technical support from the Vietnam Administration of AIDS Control and WHO. Trained peer educators collected information from clients using a client information form, which included information on age, gender, risk group, history of HIV testing, testing options, testing results, linkage to ART and the reasons for accessing community‐led testing with a closed‐ended questionnaire. Clients’ information forms and logbooks were kept in a secured cabinet at testing sites. Synthesized data were reported to the PAC and sent to the designated responsible officer every month after removal of all personal identifiers, such as names and addresses of the clients, to enhance confidentiality and anonymity. Verbal consent for the study was obtained by peer educators. The pilot was implemented according to the plan approved by the Ministry of Health (Decision 4306/QD‐BYT) 30. Since these activities were conducted as part of a public health programme evaluation, the study was not considered to be research on human subjects.

Descriptive analysis was conducted using STATA 11.2 (StataCorp, College Station, TX, USA), and included analysis of client demographics and characteristics, uptake, testing approach, testing history, age, sex, key population group and risk behaviours. All data on aPN were analysed and described separately.

## 3 Results

### 3.1 HIV testing uptake and clients’ characteristics

From January 2017 to May 2018, community‐led HTS delivered HIV testing to 3978 persons from key populations and their partners (22.4% opted for self‐testing and 77.6% opted for lay provider testing). Nearly all those tested were male (93.7%) and nearly half (44.4%) were between the ages of 16 and 25 years. The majority of those tested self‐identified as MSM (54.9%) or as PWID (39.0%). The remaining were FSWs (3.9%), and partners and children of PLHIV (2.0%). A small proportion identified as coming from another high‐risk group, for example, truck drivers, mobile workers, or group not reported (Table 1). The number of people who refused testing was not recorded since various channels were used to promote and create a demand for the services, including Facebook and other social networks, as well as conventional outreach. As peer educators had difficulty in tracking refusal information, only those who came for testing were recorded.

**Table 1:** Clients’ characteristics (self‐reported)

	Can Tho n (%)	Thai Nguyen n (%)	Total N (%)
Gender			
Male	941 (85.3)	2786 (96.9)	3727 (93.7)
Female	129 (11.7)	89 (3.1)	218 (5.5)
Transgender male	1 (0.1)	0	1 (0.03)
Transgender female	32 (2.9)	0	32 (0.8)
Age group			
18 months to 15 years	0	4 (0.1)	4 (0.1)
16 to 25 years	653 (59.2)	1112 (38.7)	1765 (44.4)
26 to 49 years	410 (37.2)	1737 (60.4)	2147 (54.0)
>49 years	40 (3.6)	21 (0.7)	63 (1.5)
Unknown	0	1 (0.03)	1 (0.03)
Risk group			
PWID	0	1551 (54.0)	1551 (39.0)
FSW	129 (11.7)	24 (0.8)	153 (3.9)
MSM	950 (86.1)	1235 (43.0)	2185 (54.9)
Partners of PLHIV	23 (2.1)	54 (1.9)	77 (1.9)
Children of PLHIV	0	4 (0.1)	4(0.1)
Other^a^	1 (0.1)	7 (0.2)	8 (0.2)
Total	1103	2875	3978

^a^Others: long distance drivers, mobile workers, and those in whom no specific risks were reported. PWID, people who inject drugs; FSW, female sex worker; MSM, men who have sex with men; PLHIV, people living with HIV.

Among clients receiving HTS, 2654 (66.7%) reported that they had never tested for HIV previously. Among 1264 clients who had ever been tested before, 46% reported being tested more than a year ago (Table 2). Among those previously tested, two had been diagnosed as HIV positive and were on ART and were excluded from the HIV cascades analysis.

**Table 2:** HIV testing history

	Can Tho n (%)	Thai Nguyen n (%)	Total N (%)
HIV testing (N = 3978)			
Ever tested	511 (46.3)	753 (26.2)	1264 (31.8)
Never tested	592 (53.7)	2062 (71.7)	2654 (66.7)
Don't remember	0	60 (2.1)	60 (1.5)
Time since last test (n = 1264)			
≤12 months	437 (85.5)	241 (32.0)	678 (53.6)
>12 months	69 (13.5)	512 (68.0)	581 (46.0)
Unknown	5 (1.0)	0	5 (0.4)
Test results (n = 1264)			
Positive	2 (0.4)	0	2 (0.2)
Negative	502 (98.2)	658 (87.4)	1160 (91.8)
Don't remember	7 (1.4)	95 (12.6)	102 (8.1)

Among those asked why they chose community‐led HTS versus other approaches (N = 3978), the most common reasons were: hesitation to go to a health facility (59.5%), ensured confidentiality of results (52.6%), convenience (62.8%) and services being free of charge (61.3%). Responses from those opting for lay provider‐delivered HTS or self‐testing were similar. Responses of those reached via aPN were also similar, except for preferring testing due to services being free and fewer preferring confidential results (Table 3).

**Table 3:** Reasons for accessing community‐led HIV testing by lay provider and self‐testing

Reasons	Lay testing (%) n = 3086	Self‐testing (%) n = 892	Total N = 3978	Partner notification^a^ (%) n = 105
Hesitation to go to health facility	1779 (57.7)	588 (65.9)	2367 (59.5)	50 (47.6)
Ensured confidentiality of the results	1677 (54.3)	416 (46.6)	2093 (52.6)	21 (20.0)
Convenience	2000 (64.8)	496 (55.6)	2496 (62.8)	47 (44.8)
Services free of charge	1825 (59.1)	615 (69.0)	2440 (61.3)	78 (74.3)
Comfortable	6 (0.2)	3 (0.3)	9 (22.6)	11 (10.5)

^a^This data was also included in lay and self‐testing.

### 3.2 Effectiveness of lay provider testing, self‐testing

**Figure 3:**
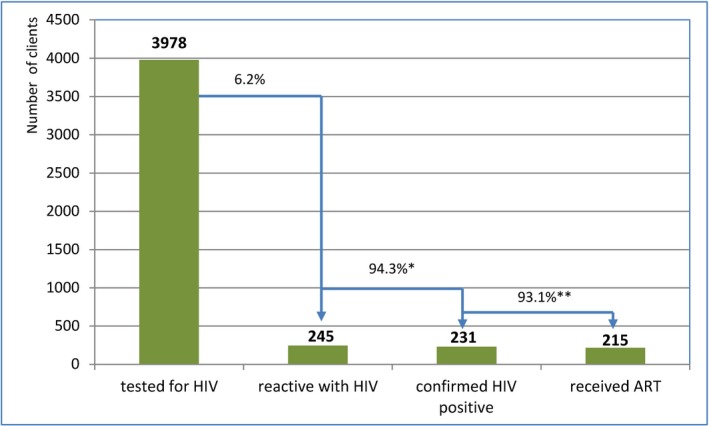
**Overall HIV cascades from two provinces.** *False reactive – 3; lost to follow‐up – 4; not yet gone for confirmatory tests – 5, already on antiretroviral therapy (ART) – 2. **Died – 1, not yet gone for ART – 6, moved to other provinces – 2, lost to follow‐up – 2.

Community‐led HTS plus aPN reached a large proportion of people with HIV. Of those tested, 6.2% (245/3978) had a reactive result and 5.8% (231/3978) were confirmed to have HIV. There was a significant difference in HIV prevalence among key population groups (χ^2^ = 21.6; *P* < 0.001). HIV reactivity was highest among partners of PLHIV receiving HTS (28.6%), followed by MSM (7.3%), FSWs (5.2%) and PWID (3.3%) (Table 4). The majority of those with a reactive test result were linked to confirmatory testing (95.5%, 234/245) including three clients confirmed to have false‐reactive results, and initiated on treatment (93.1%, 215/231) (Figure 3).

**Table 4:** HIV cascades by key populations, including assisted partner notification

	Tested for HIV	Reactive to HIV rapid test^a^		Confirmed HIV positive		Received ART	
	N	n	%	n	%	n	%
Key populations		χ^2^ = 27.8; *P* < 0.0001		χ^2^ = 21.6; *P *< 0001		χ^2^ = 0.02; *P *= 0.98	
PWID	1551	51	3.3	51	100	47	92.2
FSW	153	8	5.2	7	87.5	7	100.0
MSM	2185	160	7.3	147	91.9	137	93.2
Partners^a^ of PLHIV previously diagnosed	77	22	28.6	22	100.0	22	100.0
Children of PLHIV	4	2	50.0	2	100.0	1	50.0
Others^b^	8	2	25.0	2	100	1	50.0
Choice of testing		χ^2^ = 2.4; *P* = 0.1243		χ^2^ = 3.3; *P* = 0.07		χ^2^ = 0.003; *P* = 0.96	
Lay provider testing	3086	179	5.8	167	93.3	156	93.4
Self‐testing	892	65	7.2	63	96.9	59	93.6

^a^Including sexual and drug injecting partners; ^b^long distance drivers, mobile workers, or not reported. PWID, people who inject drugs; FSW, female sex worker; MSM, men who have sex with men; PLHIV, people living with HIV; ART, antiretroviral therapy.

**Figure 4:**
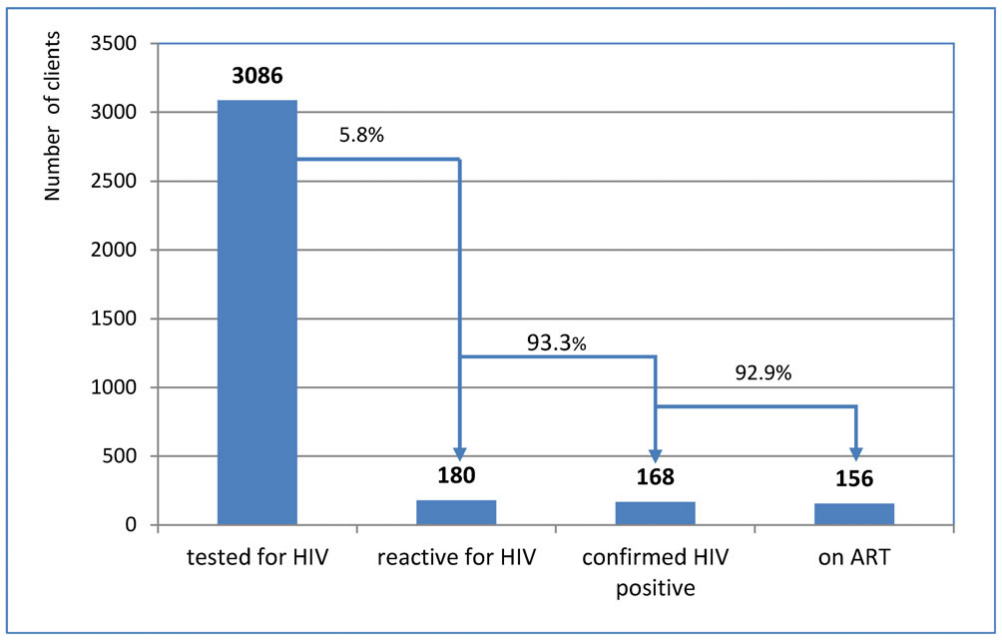
**HIV cascades by lay provider testing.** ART, antiretroviral therapy.

**Figure 5:**
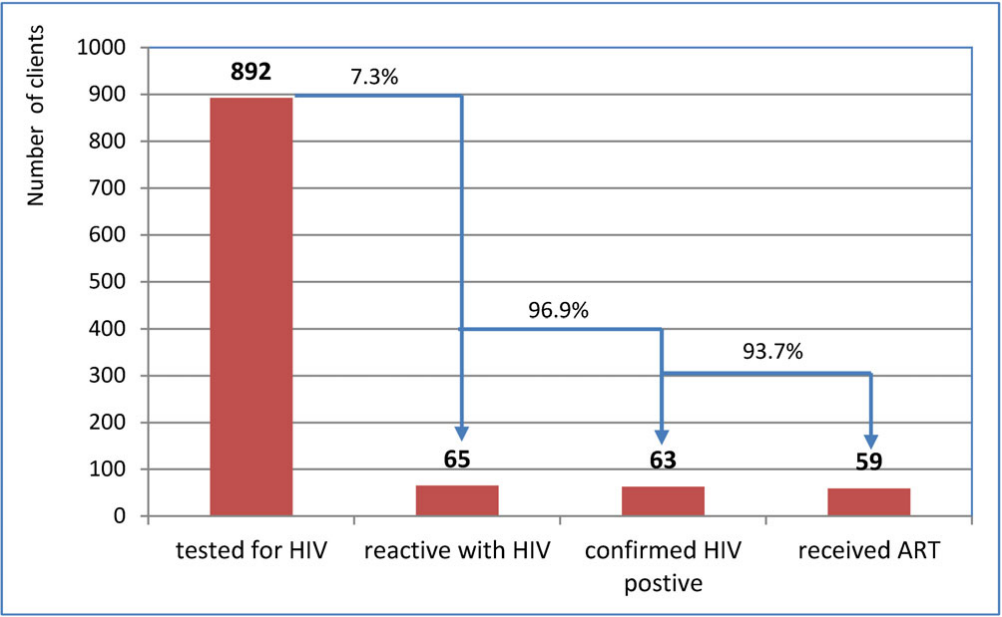
**HIV cascades among those self‐testing for HIV.** ART, antiretroviral therapy.

The proportion linked to confirmatory testing and who initiated ART was similar when comparing lay provider testing to self‐testing, and by key population, with the exception of FSWs (Table 4). However, the proportion diagnosed with HIV was higher among those self‐testing (7.3%) compared to those receiving lay provider testing (5.8%) (Figures 4 and 5).

### 3.3 aPN services

**Figure 6:**
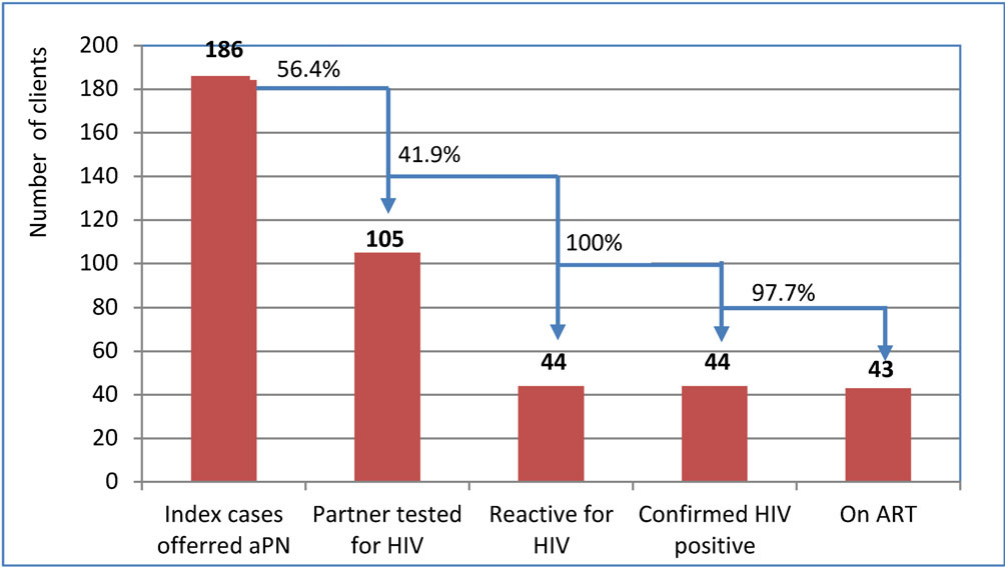
**HIV cascades among partners of HIV‐positive clients, who offered aPN.** aPN, assisted partner notification; ART, antiretroviral therapy.

Between July 2017 and May 2018, aPN was offered to 207 HIV‐positive clients, of whom 186 accepted and provided contact information, and 105 partners of these were contacted and provided with HIV testing. Most HIV‐positive clients opted for provider referral over client or dual referral because they did not want to disclose their HIV status. Using these methods, 56.4% (105/186 were successfully contacted) received HTS. The ratio of partners who tested for HIV per index client was 0.56. Forty‐four (41.9%) partners of HIV‐positive clients tested were diagnosed with HIV; 97.7% (all but one) initiated ART during the study period (Figure 6). Most partners of HIV‐positive clients who were tested identified as MSM (88/105; 83.8%) and were between 16 and 25 years of age (57/105; 54.2%) (Table 5). Several challenges were reported in implementing aPN among key populations and their partners. PLHIV clients who refused aPN were asked why and reported that they were afraid to disclose their status to their partners, and many also reported that they did not have sufficient contact information for casual partners, for example, “one‐night stands.” All partners who tested HIV negative were provided with condoms and needles–syringes, based on their needs.

**Table 5:** Characteristics of assisted partner notification

	Index clients N	Partners of index clients receiving HIV tests n (%)	Partners who had HIV‐positive results n (%)
Age group (years)			
16 to ≤ 25	57	57 (100)	27 (47.4)
>25	128	48 (37.5)	17 (35.4)
Not reported	1	0	0
Risk group			
PWID	50	1 (2.0)	1
FSW	6	0	0
MSM	110	88 (80.0)	37 (42.0)
Sexual partners	18	16 (88.9)	6 (37.5)
Others	2	0	0
Total	186	105 (56.5)	44 (41.9)

PWID, people who inject drugs; FSW, female sex worker; MSM, men who have sex with men.

### 3.4 Social harm

Since January 2017, two cases with suicidal thoughts were reported after an initial HIV‐positive diagnosis. However, these cases received timely social support from peer educators and both clients were enrolled in care and treatment. No social harm was identified or reported during implementation of community‐led HTS with regard to lay provider testing, self‐testing or aPN.

## 4 Discussion

The results of our study provide strong evidence that delivering aPN as part of a package of community‐led HTS with lay provider and self‐testing is acceptable and feasible among key populations in two provinces in Vietnam. After more than one year of community‐led HTS plus aPN, almost 4000 persons from key populations and their partners were tested, and 231 were diagnosed to be HIV positive. While other studies have indicated that peer‐led interventions, including aPN, increase the uptake of HIV testing among MSM 31, 32, our results reflected outcomes among other key population groups, and the impact of implementing aPN as a part of a comprehensive community‐led HTS package. Similar to other studies, our findings show a high uptake of HIV testing, and good linkage to care and treatment 33. The dedication of peer educators in supporting clients with reactive results to access confirmatory testing and ART services is thought to have contributed to the linkage results. Data are limited on lay provider testing and self‐testing among PWID, FSWs and partners of PLHIV but previous studies on HIV self‐testing among MSM show lower but still impressive linkage, for example, 77.5% in China and 78% in the United Kingdom 34, 35.

Our results confirm previous findings that show aPN to be a highly effective way of identifying new HIV‐positive cases and facilitating ART initiation 36, 37, 38, 39. According to the Viet Nam Authority for HIV/AIDS Control, in 2017, the annual proportion of people testing positive was 2.7% among key populations 40. Implementing community‐led HTS among key populations and their partners in our study was significantly higher (5.8%), largely due to aPN, which was the most effective case‐finding strategy employed by peer educators. More than 40% of partners of PLHIV tested through aPN were diagnosed with HIV and nearly all were linked to treatment. However, although the impact of aPN reported in this study is compelling, only 44 HIV‐positive cases were identified through this approach. To have a public health impact, aPN among key populations and their partners must be scaled up. Additional strategies to scale‐up and increase effectiveness need to be considered and introduced in community outreach for key populations. We experienced similar challenges as reported by Dalal et al. 7 in eliciting and contacting partners of PLHIV, particularly in reaching casual partners. Additional efforts are needed to identify and implement the best ways to reach these partners and deliver timely HTS.

The ratio of partners tested per index case in our pilot (0.56) was relatively lower than that reported in a systematic review (0.85 (0.19 to 1.81)) 7. Our data, also could not determine the proportion of new HIV infections among partners infected with HIV. Previous studies have suggested that aPN may play a key role in early identification of HIV‐positive partners, as well as prevention of HIV transmission. According to an observational study by Green et al. 36% of partners tested for the first time through aPN had acute or early HIV infection 41. We did not attempt to detect acute infection or use recency testing. However, 17 out 44 (38.6%) HIV‐diagnosed partners had an HIV negative test in the past year (data not shown) and these cases could be new infections. Future efforts to scale‐up aPN in Vietnam should consider how to not only improve case‐finding but also prevent new infections in key populations, such as linking HIV‐negative partners and clients to pre‐exposure prophylaxis.

Utilizing social media and innovations such as self‐testing may have contributed to the programme's ability to reach a large proportion of young key populations and men who are more likely to be first‐time testers and at higher HIV risk. Nearly half (44.4%) of all those reached for HIV testing were young between 16 and 25 years of age (Table 1), and 66.7% of all those reached were first‐time testers (Table 2). Previous studies have suggested that social media and dating apps are appealing to young people and may increase the uptake of testing services – and also provide innovative ways of implementing aPN 6, 41, 42. Offering aPN via lay provider and self‐testing may also have increased access to and uptake of testing services among key populations, men and young people 9, 32. Our study showed that self‐testing and lay provider testing were strongly preferred to facility‐based options because it was more convenient (55.6% and 64.8% respectively) and their confidentiality was protected (46.6% and 54.3% respectively). The majority of peer educators delivering aPN and community‐led HTS were also young men from key population groups, which may have enabled the programme to reach similar groups. Future programmes should consider who they are trying to reach and select peer educators from these population groups. Strategies to use HIV self‐testing to support scale‐up of aPN and reach partners of PLHIV may be particularly useful in this setting and population 25, 26. Since July 2018, unassisted HIV self‐testing has been implemented with aPN in our programme, which could facilitate greater client uptake and referrals for partner notification.

While not the primary focus of our study, importantly, our findings confirmed that trained lay providers and self‐testers can provide accurate HIV testing results. Only three of the 3978 (0.07%) specimens were found to be false reactive on confirmatory testing. Non‐reactive specimens were not retested and therefore there is no information on the rates of false‐negative results. Although previous studies have reported 100% concordance between lay provider test results and that of medical staff 43, both the HIV RDTs used for lay provider testing and self‐testing have high specificity (99.2% to 99.9%) 44. Thus, a small proportion of false‐reactive test results are to be expected. Our study used a triage approach, where peer providers referred all those with reactive tests for further testing according to the validated national algorithm, and there was a high degree of linkage to treatment. All aPN should be conducted only after a HIV‐positive status has been confirmed, and not after reactive results to the initial test for triage. Programmes implementing community‐led HTS using an initial test for triage need to ensure that they deliver the correct messages and achieve good linkage to onward services.

During the study period, there were two cases of suicidal ideation. Both cases were quickly identified and addressed by trained peer educators, which prevented potential harm from escalating. No other social harm such as self‐harm, suicide or intimate partner violence was identified or reported during the 17‐month study period. This is consistent with other studies on lay provider testing, self‐testing, and aPN, showing that such events are rare 7, 45, 46. Despite these encouraging findings, it is critical that programmes sensitize communities, health workers, and prepare and train peer educators to provide the necessary support and messages, as well as tools to monitor and report on social harm. Such approaches will continue to be needed as implementation proceeds and is scaled up beyond the pilot phase.

Our study has several limitations. First, as this was an operational research study, data collection was kept to a minimum to both reflect a real‐world setting and ensure correct and complete data capture. A full evaluation would be needed to provide more comprehensive information, including qualitative data on the facilitators of and barriers to HIV lay provider testing, self‐testing and partner notification services. Second, interventions were introduced in a step‐wise manner; thus it was not possible to say which testing service (lay provider vs. self‐testing) was preferred by key populations. Third, there were only two peer educators who were FSWs. This likely resulted in fewer clients being reached from this key population.

This project generated country evidence for national policy development. The findings from the pilot programme informed development of national guidelines for community‐based testing, including lay provider and self‐testing, and aPN. The guidelines were approved by the Ministry of Health in 2018 47 and these approaches were scaled up in other high‐burden provinces.

## 5 Conclusions

As part of community‐led HTS, aPN is an effective and feasible HIV case‐finding strategy for key populations and can contribute to reaching a large number of young male key populations in Vietnam. Efforts are needed to scale up aPN alongside community‐led HTS – including lay provider and self‐testing – to achieve a public health impact. Further research is needed on how to improve the reach of aPN services among casual and non‐primary partners.

## Competing interests

The authors have no competing interests to declare.

## Authors’ contributions

VTTN designed the pilot, planned and monitored the implementation, provided technical guidance for data analysis and led the writing of the manuscript. PTTH and MK provided technical inputs for planning and implementation, and reviewed the manuscript; VHS, PQT and LAKA supervised and provided technical support to the implementation and inputs to the development of the manuscript. DCT assisted with data analysis and review of the manuscript, RB provided technical inputs during implementation and reviewed the manuscript. CJ provided technical inputs for planning and implementation, and assisted with manuscript development.
